# Molecular and Cellular Foundations of Aging of the Brain: Anti-aging Strategies in Alzheimer’s Disease

**DOI:** 10.1007/s10571-024-01514-0

**Published:** 2024-11-28

**Authors:** Magdalena Dziewa, Magdalena Złotek, Mariola Herbet, Iwona Piątkowska-Chmiel

**Affiliations:** Chair and Department of Toxicology, Faculty of Pharmacy, Medical University of Lublin, Jaczewskiego 8b Street, 20-090 Lublin, Poland

**Keywords:** Cellular aging, Anti-aging interventions, Alzheimer’s disease, Lifestyle interventions, Gene therapy, Cell based-therapy

## Abstract

Alzheimer’s disease (AD) is a condition characterized by the gradual degeneration of the nervous system that poses significant challenges to cognitive function and overall mental health. Given the increasing global life expectancy, there is an urgent need for effective strategies to prevent and manage Alzheimer’s disease, with a particular focus on anti-aging interventions. Recent scientific advancements have unveiled several promising strategies for combating Alzheimer’s disease (AD), ranging from lifestyle interventions to cutting-edge pharmacological treatments and therapies targeting the underlying biological processes of aging and AD. Regular physical exercise, cognitive engagement, a balanced diet, and social interaction serve as key pillars in maintaining brain health. At the same time, therapies target key pathological mechanisms of AD, such as amyloid-beta accumulation, tau abnormalities, neuroinflammation, mitochondrial dysfunction, and synaptic loss, offering potential breakthroughs in treatment. Moreover, cutting-edge innovations such as gene therapy, stem cell transplantation, and novel drug delivery systems are emerging as potential game-changers in the fight against AD. This review critically evaluates the latest research on anti-aging interventions and their potential in preventing and treating Alzheimer’s disease (AD) by exploring the connections between aging mechanisms and AD pathogenesis. It provides a comprehensive analysis of both well-established and emerging strategies, while also identifying key gaps in current knowledge to guide future research efforts.

## Introduction

Senescence is a natural and inevitable process that occurs in all organisms, preventing the proliferation of damaged cells and playing a role in embryonic development and tissue repair. However, cellular senescence is also linked to not only cancer, but also various age-related diseases, such as hypertension, Alzheimer's, Parkinson's, and more (Calcinotto et al. 2019). In today's rapidly evolving world, characterized by increasing life expectancies globally, the significance of senescence becomes ever more apparent. Aging, an intrinsic and inevitable process, heralds a cascade of physiological and psychological transformations that intricately shape an individual's existence. These changes, from declining physical prowess to cognitive shifts, profoundly influence the quality of life and one's ability to navigate the complexities of daily life autonomously. Among the myriad challenges entwined with aging, AD emerges as a particularly formidable foe (Hebert et al. 2013).

Alzheimer's disease (AD) is a progressive neurodegenerative disorder, poses a significant threat to cognitive function, memory retention, and overall mental acuity. Its relentless advance compromises individual autonomy and places immense strain on caregivers and healthcare systems worldwide (Calcinotto et al. 2019). On a global scale, nearly 50 million individuals grapple with dementia, and given the ongoing demographic transition towards an aging society, this figure may triple in the foreseeable future. Statistics reveal a discernible, that 3% of individuals aged 65–74 are affected by AD, a figure that surges to 17% among those aged 75–84. The prevalence further escalates to 32% among individuals aged 85 and older. Notably, Alzheimer's dementia can, albeit less frequently, also afflict individuals under the age of 65, though the prevalence in this demographic remains uncertain (Hebert et al. 2013). Moreover, the prevalence of AD exhibits regional disparities, with Europe and North America reporting higher rates compared to Asia, Africa, and South America (Cao et al. 2020). These variations could stem from genetic predispositions, lifestyle factors, and environmental influences, necessitating comprehensive investigations to better understand and address the disease's global impact. In Europe, AD exhibits an estimated prevalence of approximately 5.05% (95% CI 4.73–5.39), emphasizing its substantial impact on a significant portion of the population (Niu et al. 2017). Notably, the incidence of AD in men is lower, estimated at 3.31% (95% CI 2.85–3.80), while in women, it markedly escalates to an estimated prevalence of 7.13% (95% CI 6.56–7.72) (Niu et al. 2017). This disparity in Alzheimer's prevalence between genders underscores the intricate interplay of biological and environmental factors.

This alarming trend emphasizes the urgent need to confront the intricate challenges and profound impact of AD within the context of an increasingly aging population. The multifaceted nature of AD poses a significant hurdle in the pursuit of a definitive solution (Cao et al. 2020). Despite notable progress in unraveling the underlying mechanisms of AD, translating this understanding into an effective cure remains an arduous challenge. Researchers worldwide persist in their unwavering dedication, driven by a shared commitment to alleviate the profound suffering inflicted by this devastating disease and enhance the quality of life for those affected and their families (Cao et al. 2020). AD exacts a profound toll on individuals and communities alike. From the personal anguish experienced by those directly affected to the broader socioeconomic ramifications felt across society, the ripple effects of AD are far-reaching and demand concerted efforts to mitigate its devastating effects.

**Fig. 1:**
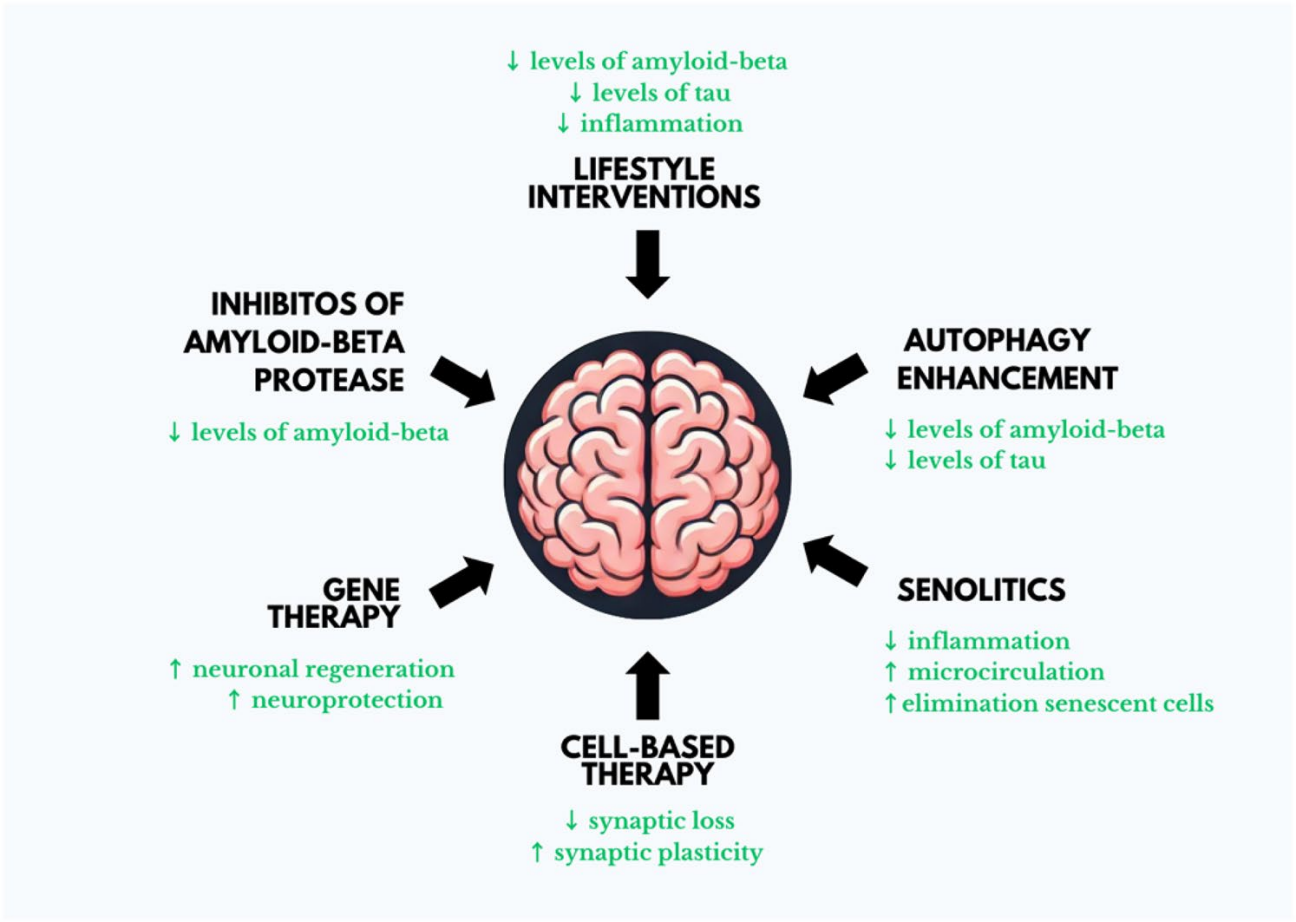
Innovative approaches to mitigating Alzheimer's disease progression. Exploring cutting-edge strategies such as lifestyle modifications, advanced gene therapies, pharmacological advancements, and cell-based treatments to enhance cognitive function, delay disease progression, and improve overall quality of life for Alzheimer's patients

This review delves into the pivotal role of cellular aging in the progression of Alzheimer's disease (AD) and assesses a variety of interventions designed to address these interconnected processes. Cellular aging, marked by telomere shortening, mitochondrial dysfunction, and the accumulation of senescent cells, significantly contributes to the pathogenesis of AD. These age-related changes foster neuroinflammation and synaptic degeneration, which in turn accelerate cognitive decline. Special attention is given to lifestyle modifications, such as nutrition and physical activity, that have demonstrated efficacy in enhancing cellular health and potentially alleviating the impacts of aging. In addition, we conduct a analysis of both pharmacological interventions and cutting-edge therapies that specifically target the key mechanisms linking the aging process to the pathology of Alzheimer's disease. By investigating these innovative approaches, we aim to elucidate how they may mitigate the detrimental effects of cellular aging and provide therapeutic benefits for AD patients (Fig. 1).

## Materials and Methods

The following databases were utilized for a comprehensive literature review: PubMed, Scopus, and Web of Science. The search terms included "Alzheimer's disease," “Aging”, "Anti-aging interventions," "Cell therapy," "Gene therapy," and "Lifestyle interventions." The search was restricted to human subjects, English language, peer-reviewed articles, and publications from the last 15 years, utilizing Medical Subject Headings (MeSH) terms and Boolean operators. To ensure a rigorous selection process, specific inclusion criteria were meticulously applied, targeting studies that directly addressed the relationship between aging and Alzheimer's disease (AD). This review primarily focused on adults diagnosed with AD, delving into the molecular changes associated with cellular aging and their implications for the disease's progression. It also evaluated the impact of lifestyle interventions and pharmacotherapy on cognitive function and brain volume in individuals with AD, highlighting the importance of holistic approaches to treatment. The review encompassed a variety of study types, including clinical trials, literature reviews, cohort studies, cross-sectional studies, and meta-analyses, all of which aligned with its objectives of understanding the multifaceted nature of AD and aging. To maintain a focused analysis of pertinent research findings, the inclusion criteria restricted the examination to studies published exclusively in English. This ensured that the review reflected the most relevant and accessible literature, providing valuable insights into the interplay between aging and AD and identifying potential avenues for future research and therapeutic interventions.

## Relationship Between Cellular Aging and Alzheimer's Disease

**Fig. 2:**
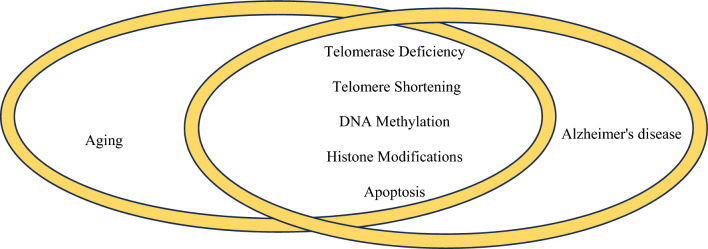
Similarities in the molecular changes of cell aging and Alzheimer's disease

Cellular aging and AD share several molecular changes, reflecting common pathways and mechanisms that contribute to the decline in cellular function and the progression of neurodegenerative conditions. Both processes involve alterations in protein homeostasis, mitochondrial dysfunction, oxidative stress, and inflammation. Additionally, they are characterized by telomerase deficiency, telomere shortening, DNA methylation changes, histone modifications, and increased apoptosis (Fig. 2).

### Molecular Changes Associated with Cellular Aging and Alzheimer's Disease

#### Telomerase Deficiency and Telomere Shortening

Telomeres, those protective caps found at the tips of chromosomes, consist of genetic material and undergo gradual shortening with each cell division as part of the natural aging process. Their length varies across species, ranging from 10 to 15 kb in humans (de Lange et al. 2002). Serving as a biological clock, their length often correlates with chronological age. When telomeres reach a critically short length or become dysfunctional, they can trigger a response akin to DNA damage, prompting cells to either cease division and enter a state of senescence or undergo programmed cell death, termed apoptosis (Kong et al. 2013). However, beyond mere markers of time passage, telomere shortening has emerged as a contributing factor to the onset and progression of neurodegenerative conditions associated with aging, including AD (von Zglinicki 2002; Boccardi et al. 2015).
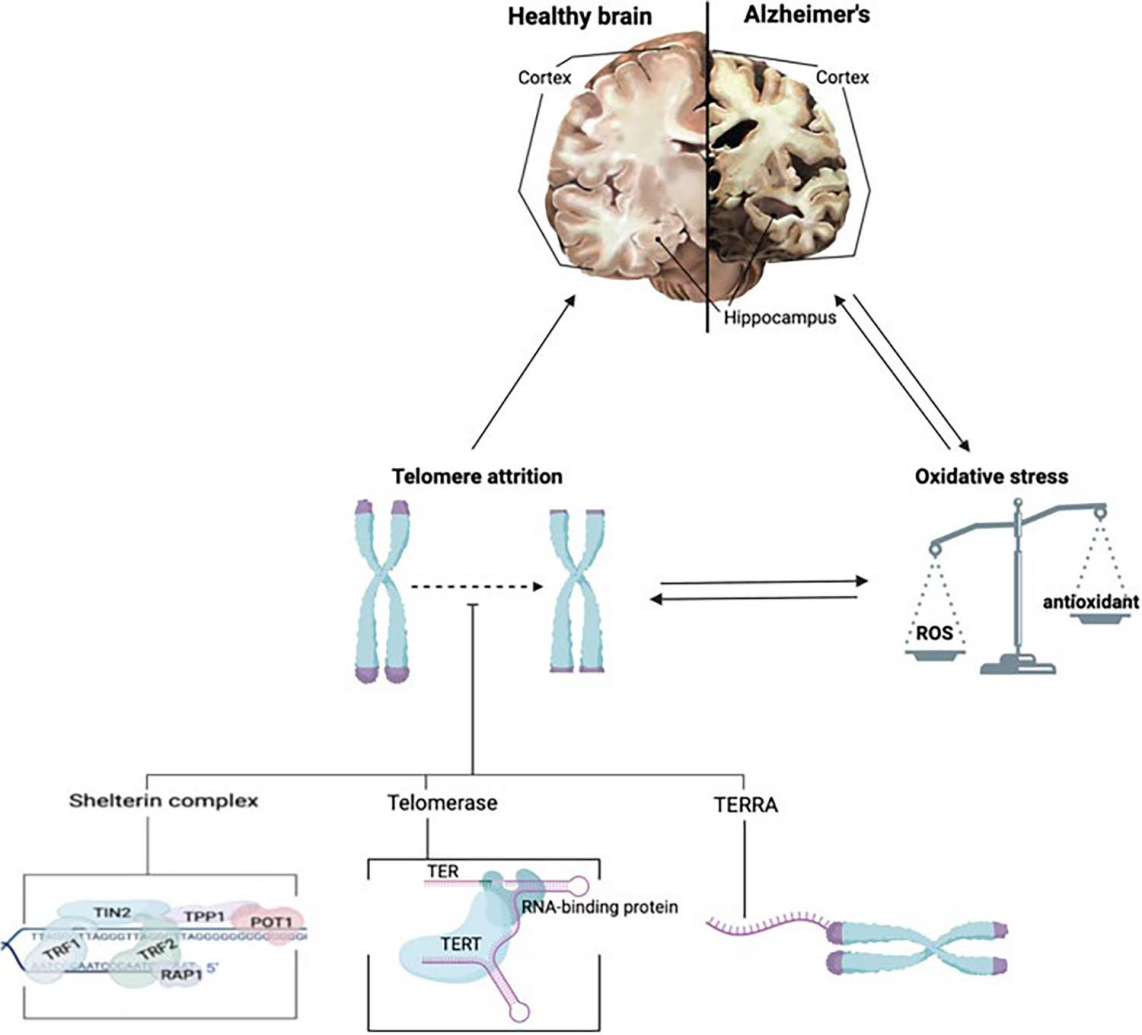


Groundbreaking insights from Cai et al. (2013) have illuminated the central role of telomere attrition in the pathogenesis of AD, underscoring the intricate relationship between cellular aging mechanisms and neurodegeneration. The gradual shortening of telomeres in leukocytes and microglia may indirectly impact neuronal health by disrupting the normal functions of these immune cells within the brain (Weng 2012; Rolyan et al. 2011). Nevertheless, the precise extent to which telomere erosion contributes to the progression of Alzheimer's remains elusive, owing to inconsistencies among research findings.

#### DNA Methylation

DNA methylation predominantly takes place on cytosine residues within cytosine-guanine (CpG) dinucleotides in mammals, serving to regulate gene expression (Yokoyama et al. 2017).

A group of DNA methyltransferases, with DNMT1, DNMT3A, and DNMT3B being the most extensively studied (Handel et al. 2010), are the cause of the methylation process. These enzymes catalyze the transfer of a methyl group onto single-stranded DNA, utilizing S-adenosyl methionine as the methyl donor. DNMT1 is particularly crucial for maintaining methylation in somatic cells, and its deficiency has been linked to nuclear disorganization, heightened histone acetylation, and cell apoptosis (Mastroeni et al. 2010). Notably, an increase in plasma homocysteine levels, a component of this metabolic pathway, is correlated with an increased risk of dementia and AD, thus suggesting a connection between DNA methylation, alterations in one-carbon metabolism, and the pathogenesis of AD (Yokoyama et al. 2017).

DNA methylation, especially the presence of 5-methylcytosine (5mC), is a key epigenetic change found in the brains of Alzheimer's disease (AD) patients (Chouliaras et al. 2010). As the brain ages, levels of 5mC rise (Chouliaras et al. 2012) which may affect to synaptic plasticity, learning, and memory process.

Recently, attention has shifted to 5-hydroxymethylcytosine (5hmC), a derivative of 5mC (Dahl 2011). 5hmC is found in all examined tissues, including the brain, liver, and spleen, with low levels in stem cell-rich areas and higher levels in fully differentiated neurons (Coppieters et al. 2014; Chouliaras et al. 2012). 5hmC may also be involved in diseases like AD, as certain genes expression related to ion transport, neuronal development, and cell death correlation with higher levels of 5hmC at specific regions of brain (Coppieters et al. 2014; Chen et al. 2012).

#### Histone Modifications

Beyond DNA methylation, disruptions in histone modifications stand out as significant epigenetic alterations implicated in the pathogenesis of AD. Histone posttranslational modifications (hPTMs) affect the DNA-associated proteins forming the core histone octamer, thereby influencing chromatin structure dynamics (Fenoglio et al. 2018). These modifications play a critical role in epigenetic regulation by either altering chromatin accessibility through their strong interaction with DNA or by recruiting other proteins to specific DNA regions (Pal and Tyler. 2016).

The N-terminal tails of histones boast multiple sites susceptible to posttranslational modifications, including methylation, acetylation, phosphorylation, ubiquitylation, and SUMOylation (Hwang et al. 2017). Among these, histone acetylation stands out as an extensively studied posttranslational modification. It involves the addition of acetyl groups, sourced from the cofactor acetyl CoA, to specific lysine residues within the N-terminal tails of histones, notably lysines on H3 and H4. This modification cancels out the positive charge of lysines, thereby weakening the interaction between DNA and histones. As a result, it enhances accessibility for the transcription apparatus to gene promoters. (Hwang et al. 2017; Bannister et al. 2011). Moreover, histone acetylation plays a pivotal role in recruiting transcription factors (TFs) to promoter regions, thereby enhancing their binding (Bannister et al. 2011). In the human brain, numerous processes hinge on histone acetylation, including memory formation, consolidation, and synaptic plasticity. In the hippocampus, histone acetylation is particularly involved in forming long-term memories and excitatory synapses, which are essential for different types of synaptic plasticity like long-term potentiation (LTP) (Geng et al. 2021).

Histone phosphorylation, much like acetylation, is strongly linked to active transcription, facilitating the relaxation of chromatin structure by neutralizing the positive charge of histones. This modification involves the incorporation of a phosphate group to serines, tyrosines, and threonines by histone kinases (Bannister et al. 2011). In brain function, histone phosphorylation plays a role in pathways pertinent to memory formation and the transcriptional regulation of immediate-early genes, which serve as markers (Santana et al. 2023).

#### Apoptosis

Apoptosis, often dubbed as "programmed cell death," is a meticulously orchestrated process regulated by genes, orchestrating the controlled and typically painless demise of cells. It functions as a natural mechanism for eliminating damaged, aging, or superfluous cells in multicellular organisms, playing a pivotal role in the development and maintaining tissue equilibrium. During apoptosis, cells exhibit notable morphological changes, including shrinkage, nuclear condensation, DNA fragmentation, and the formation of apoptotic bodies, culminating in cell disintegration without provoking inflammation (Elmore 2007).

The aging process disrupts the delicate balance between pro-apoptotic and anti-apoptotic factors, contributing to a gradual decline in organ function. In the brain, increased neuronal apoptosis has been associated with cognitive decline, even in individuals who do not exhibit clear signs of neurodegenerative diseases (López-Otín et al. 2013). Additionally, the accumulation of apoptotic cells can trigger inflammatory responses, which further exacerbate tissue damage and promote additional cell death. With advancing age, there is a notable increase in reactive oxygen species (ROS) coupled with a decline in antioxidant defenses, leading to significant neuronal loss. Moreover, aging is characterized by mitochondrial dysfunction and reduced energy production, which can facilitate the release of pro-apoptotic factors, such as cytochrome c, into the cytosol, thereby promoting the apoptotic process (Goel et al. 2022).

**Fig. 3:**
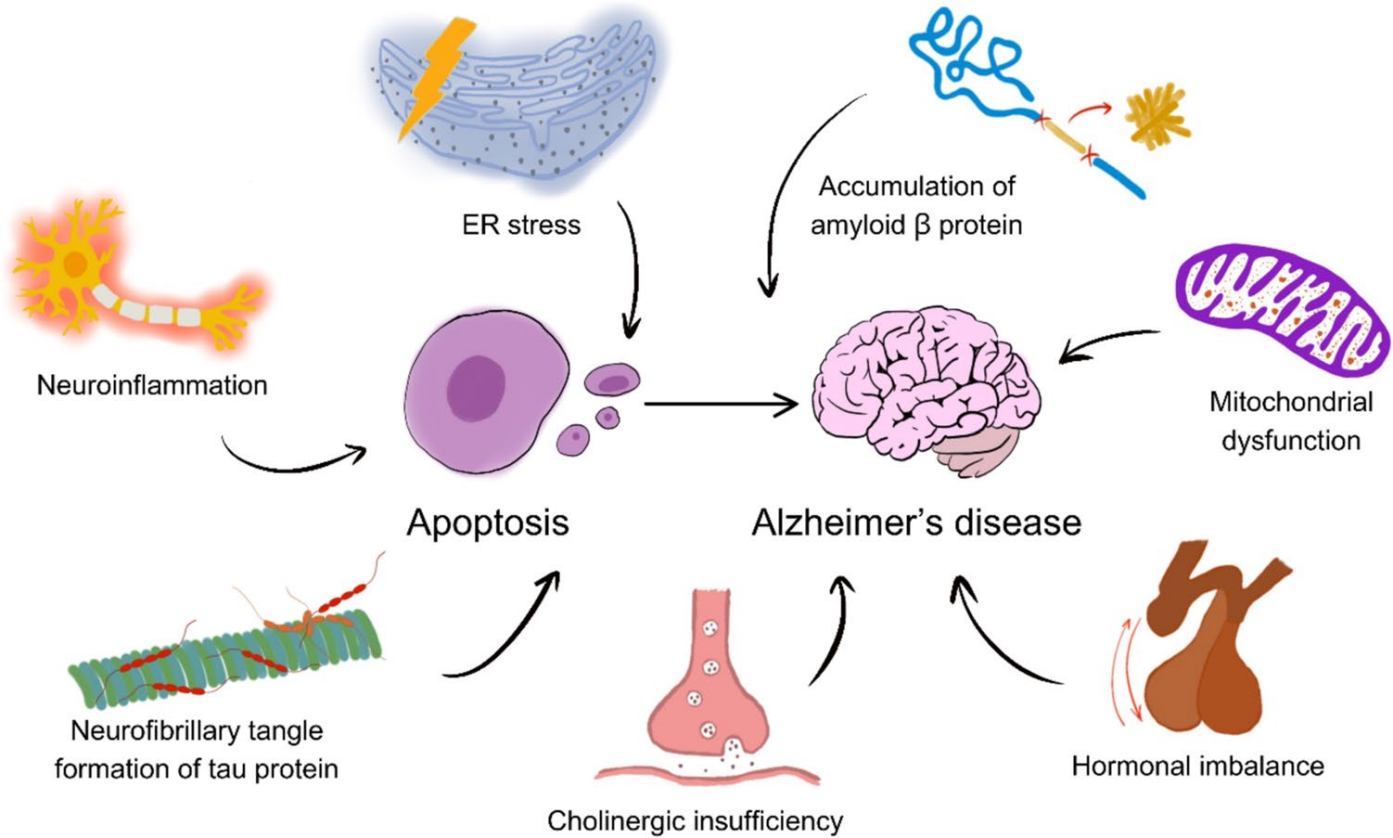
Pathogenetic factors of apoptosis in Alzheimer's disease

In the context of AD, extensively studied pathological hallmarks such as amyloid-beta plaques, tau protein tangles (NFTs), inflammation, mitochondrial dysfunction, oxidative stress, and excitotoxic cell death instigate an aberrant apoptotic process primarily in vulnerable brain regions like the cerebral cortex and hippocampus (Goel et al. 2022) (Fig. 3). The culmination of this apoptotic cascade results in abnormal neuronal loss, representing a primary event that may precede other aspects of AD progression and strongly correlates with the severity of dementia (Sharma et al. 2021). Upon closer scrutiny of the initial stages of AD pathology, it has been recognized that significant neuronal loss in AD predominantly occurs through apoptosis, intertwining with key pathological markers of the disease (Shimohama 2000).

Regardless of the initial triggers, neuronal demise in AD can manifest through either apoptosis or necrosis, contingent upon the strength and persistence of the stimuli. Necrosis brings about swift and irreversible cell demise once initiated, while apoptosis unfolds as a more regulated process, prompted by milder stimuli (Sharma et al. 2021). In AD patients, neuronal loss precipitates the depletion and damage of hippocampal cells, profoundly impacting attention and cognitive functions governed by the hippocampus (Obulesu et al. 2014).

Apoptosis intricately collaborates with numerous critical factors in AD pathology, encompassing both pro-apoptotic and anti-apoptotic elements such as B-cell lymphoma 2 (Bcl2), Bcl2-associated death promoter, amyloid precursor protein intracellular C-terminal domain, caspases, tumor necrosis factor-alpha (TNF-α), Bcl2-associated X protein (Bax), amyloid-beta (Aβ), enzyme activity, and reactive oxygen species production. These factors exert their influence, either directly or indirectly, on the progression of AD (Turunc et al. 2014).

In AD, the rate of neuronal loss surpasses that observed in normal aging and initiates before the emergence of Aβ plaques and neurofibrillary tangles (NFTs). Affected neurons are notably concentrated in regions such as the CA1 area of the hippocampus, the dentate gyrus, the subiculum, and the entorhinal cortex (Rao et al. 2022). This neuronal loss subsequently spreads to other brain regions, encompassing the cerebral cortex, olfactory bulbs, amygdala, cerebellum, and throughout the entire brain. The decline in neuronal numbers in critical regions like the CA1 area of the hippocampus and the entorhinal cortex is closely associated with memory deficits (Sharma et al. 2021). These impairments likely stem from disruptions in neuronal organization and the loss of structures vital for synaptic plasticity and normal learning processes. dentate fascia.

### Cellular Alterations in Aging and Alzheimer's Disease

#### Dysfunction of Brain Cells

AD is marked by extensive changes in different types of brain cells, such as neurons, astrocytes, and microglia. These changes collectively play a role in the development and advancement of the disease, affecting cognitive abilities and overall brain function (Baker et al. 2018; Pekny et al. 2016). In the aging human brain, microglia undergo significant functional deterioration, with approximately 40% of all cells experiencing morphological disturbances and weakening (Neumann et al. 2023). Recent studies have revealed elevated levels of proteins associated with aging, such as cell cycle repressors (p16, p53, and p21), in various cell types, including astrocytes, microglia, and neurons in the brains of AD patients (Baker et al. 2018; Pekny et al. 2016; Turnquist et al. 2016; McShea et al. 1997).

Neurons, being highly specialized cells with limited self-defense capabilities, play a crucial role in the adaptive responses of nervous tissue to damage. However, under stress conditions, neurons tend to reduce their activity to conserve energy, and prolonged stress can lead to neuronal death. Conversely, neuroglial cells exhibit increased neuroprotection and activate a defense response known as reactive gliosis in response to pathological attacks (Herdy et al. 2022; Mattson et al. 2006). Despite neurons traditionally being considered post-mitotic cells, emerging evidence suggests that they also undergo aging processes. When exposed to amyloid-β, some neurons exhibit abnormal cell cycle re-entry and are resistant to apoptosis, indicating a senescence-like phenotype (Jurk et al. 2012). This phenomenon appears to increase with age, suggesting a potential contribution to the spread of aging-related changes among neurons.

Research indicates that aging neurons may play a significant part in the dysregulation of the central nervous system through prolonged pro-inflammatory and pro-oxidative signaling mediated by mechanical DNA damage repair (DDR) signaling (Jurk et al. 2012). Increased expression of the transcription-activating protein MORF4 in neurons from AD patients suggests cell cycle re-entry akin to aging processes (Raina et al. 2001). Transcriptomic analyses of nerve cells further reveal metabolic dysfunction, elevated pro-inflammatory markers, and expression profiles of neurofibrillary tangles (NFTs) consistent with aging profiles in neurons derived from AD patients (Herdy et al. 2022; Dehkordi et al. 2021). This intricate interplay between aging-related changes in neurons and the pathogenesis of AD highlights the multifaceted nature of neurodegenerative diseases and underscores the need for targeted therapeutic interventions.

Astrocytes, crucial cells in the central nervous system (CNS), play a pivotal role in maintaining CNS homeostasis and providing essential support for neurons, promoting synapse formation, and ensuring neuronal health. However, during the aging process, astrocytes undergo a reactive transformation characterized by significant transcriptional alterations, potentially leading to a neurotoxic phenotype that poses a threat to the survival of neurons and oligodendrocytes (Verkhratsky et al. 2023). These changes aid in the brain's vulnerability to pathology, disrupting cellular function and contributing to the decline in cognitive abilities (Pekny et al. 2016).

AD is associated with impaired astrocytic function, which serves as a primary mechanism underlying neurological dysfunction. This dysfunction in neuronal signaling arises from disrupted regulation of neurotransmitter release, receptor sensitivity, and the overall strength of synaptic connections—factors that are critical for effective communication between neurons. Consequently, these it can lead to cognitive deficits and a variety of neurological disorders (Verkhratsky et al. 2023; Bhat et al. 2012; Turnquist et al. 2016; Bussian et al. 2018).

Studies by Turnquist et al. demonstrated an increased presence of p53β, a coactivator of full-length p53, and p16-positive astrocytes in the brains of AD patients. These senescent astrocytes were found to secrete various cytokines, including IL-6, recognized as an aging marker (Turnquist et al. 2016). Additionally, research by Bussian and colleagues revealed that mice with tau-related neurodegenerative disorders exhibited a significant accumulation of senescent astrocytes positive for p16 INK4A. Targeting the removal of neurofibrillary tangles (NFTs) from astrocytes through the reduction of tau phosphorylation and the modulation of the pathological signaling associated with senescent astrocytes presents a significant therapeutic opportunity (Bussian et al. 2018).

Understanding the complex role of reactive astrocytes is critical for developing innovative treatment strategies aimed at mitigating neurological dysfunction in the central nervous system (CNS) following acute injuries, as well as in the context of neurodegenerative diseases. Alzheimer's disease is marked by extensive changes in different types of brain cells, such as neurons, astrocytes, and microglia. These changes collectively play a role in the development and advancement of the disease, affecting cognitive abilities and overall brain function.

Microglia, often referred to as "resident macrophages," are specialized immune cells within the central nervous system (CNS), encompassing the brain and spinal cord. Their primary function is to orchestrate immune responses and regulate the delicate balance between pro-inflammatory and anti-inflammatory processes in the brain (Hu et al. 2021; Neumann et al. 2023). Under normal conditions, microglia play a beneficial role in supporting brain cell function. However, this relationship may deteriorate over time, leading microglia to acquire neurotoxic properties.

Studies in aged mice have revealed an increase in the production of pro-inflammatory cytokines such as IL-6, IL-1β, and TNF-α by microglia, all of which are hallmarks of senescent cells (SCs) (Hu et al. 2021). Interestingly, microglial proliferation remains active in AD. Research in APP/PS1 mice has shown that sustained proliferation of microglia promotes replicative aging phenotypes, characterized by increased senescence-associated β-galactosidase (SA-β-gal) activity, telomere shortening, and an aging-related transcriptional profile (Hu et al. 2021). Inhibiting this proliferation has been found to mitigate microglial senescence, amyloid-beta accumulation, and neural damage in AD mouse models, suggesting that unchecked microglial proliferation may exacerbate brain cell aging in AD.

Furthermore, microglia play a crucial role in myelin degradation, with the clearance of myelin debris carried out by these cells. However, this process can lead to the accumulation of lipofuscin in lysosomes, a marker of aging (Safaiyan et al. 2010). The TREM2 receptor on microglia detects various lipids, and disturbances in TREM2 function affect the Akt signaling pathway, which regulates lipid processing. Yoo et al. demonstrated that replacing dysfunctional microglia with myeloid cells from the bloodstream via hematopoietic cell transplantation restored TREM2 function and improved memory in AD-like mice with TREM2 mutations (Yoo et al. 2023). This underscores the potential of targeting microglia as a promising strategy for alleviating AD symptoms.

During aging, microglia accumulate lipid droplets and exhibit increased activity in producing reactive oxygen species (ROS) and secreting inflammatory cytokines, while their phagocytic capacity becomes limited. Genetic research indicate that genes related to lipid accumulation may contribute to neurodegenerative diseases (Neumann et al. 2023). Additionally, the accumulation of lipid droplets in microglia is associated with impaired phagocytosis and may contribute to microglial dysfunction and degeneration, which are pertinent to diseases like AD (Neumann et al. 2023; Marschallinger et al. 2020).

Further research should aim to delineate the molecular signatures that distinguish normally activated microglia from their senescent counterparts, providing insights into the intricate relationship between aging and neurodegenerative disorders.

#### Accumulation of Abnormal Proteins

Amyloid precursor protein (APP) is a crucial component present in brain cells, playing a pivotal role in the formation of amyloid-beta (Aβ). In healthy brains, Aβ is degraded by beta-secretase enzymes, resulting in the formation of soluble APP fragments. These fragments are further processed by γ-secretase-activating peptides. However, with age, the delicate balance in secretase activity is disrupted, leading to the cleavage of APP by beta and gamma-secretase enzymes. This aberrant processing yields insoluble amyloid-beta peptides (O'Brien et al. 2011).

AD and the natural aging process share molecular changes, with emerging evidence suggesting that AD may accelerate brain aging, implicating a significant role of Aβ (Montine et al. 2012). Aβ peptides exacerbate aging not only in central nervous system cells affected by AD but also in endothelial cells involved in cerebral amyloid angiopathy (Wei et al. 2016). Studies on 5 × FAD mice conducted by Wei et al. demonstrated that Aβ accumulation induces an upregulation of the aging-related marker p16, promoting cell senescence (Wei et al. 2016).

Tau protein, closely associated with microtubules, serves as the primary constituent of NFTs (Jucker et al. 2013). The accumulation of tau proteins may be linked to cellular aging and can trigger stress responses, initiating a chronic degenerative process leading to neuronal loss and brain dysfunction (Musi et al. 2018).

Musi et al. noticed a rise in the expression of NFT-related genes Cdkn1a and Cdkn2a, suggesting that stressed neurons may enter a state akin to cellular senescence. Consequently, NFTs formed in the early stages of the disease due to acute stress may initially confer neuronal protection against cell death. However, as the disease progresses, they may contribute to neurodegeneration through aging-like mechanisms, impacting the brain's bioenergetic state and elevating levels of toxic senescence-associated secretory phenotype (SASP) (Musi et al. 2018). These findings underscore the notion that NFTs can induce cellular aging, as observed in transgenic mice and postmortem human brain tissue.

#### Loss of Synaptic Connections and Neurotransmitters Imbalance

Loss of synaptic connections and abnormal balance of neurotransmitters are phenomena characteristic of both Alzheimer's disease and the aging process of the brain. These changes lead to the deterioration of communication between neurons and disturbances in signal heighten the risk of developing neurodegenerative diseases. Aging affects receptors and neurotransmitters in the brain, particularly cholinergic, glutaminergic, and catecholaminergic systems (Vinod et al. 2017).

The cholinergic pathway is responsible for cognitive processes, mood regulation, learning, and short-term memory. The cholinergic hypothesis of AD suggests that dysfunction in the cholinergic system leads to deterioration in memory and cognitive functions (Montine et al. 2012). With aging, some subtypes of nicotinic acetylcholine receptors (nAChRs) are reduced (Rogers et al. 1998). According to Seib and Martin-Villaba, decreased activation of the cholinergic system disrupts the proliferation and differentiation of neurons and affects apoptosis in the hippocampus in mice (Seib et al. 2015) Therefore, it can be inferred that preventing the reduction of nicotinic receptors in the cholinergic system may prevent cognitive deficits during aging and AD.

Norepinephrine and dopamine are neurotransmitters that play a significant role in regulating synaptic plasticity in the brain (Vinod et al. 2017). Norepinephrine regulates arousal and energy and controls circadian rhythms and working memory. Dopamine, besides influencing working memory, also regulates wakefulness, emotions, and motor functions. In age-related changes in the dopaminergic pathway in the brain, dopamine synthesis decreases, and the number of dopamine receptors D1, D2, and D3 decreases (Rieckmann et al. 2011). These impairments result in neurological symptoms linked to aging, such as restricted scope of arm movement, increased stiffness, and changes in cognitive flexibility (Vinod et al. 2017).

In aging and Alzheimer's disease (AD), synaptic connections undergo significant changes that profoundly impact cognitive functions such as learning and memory. Key features of aging neurons include the loss of neuronal arborization and reduced spine density, resulting in fewer synaptic connections (Petralia et al. 2014). Synaptic spines, which are small protrusions on dendrites where synapses form, play a critical role in facilitating synaptic transmission and plasticity. In particular, the reduction of thin spines in the prefrontal cortex (PFC), which are crucial for synaptic plasticity and learning, is notable. Similarly, there is a decline in mushroom spines in the hippocampus, which are essential for long-term memory consolidation (Petralia et al. 2014; Nicholson et al. 2004). These synaptic alterations are driven by various molecular changes. One significant factor is the reduced expression of synaptic proteins, such as postsynaptic density protein 95 (PSD-95) and synaptophysin, which are essential for maintaining synaptic structure and function. Additionally, impaired neurotransmitter signaling contributes to synaptic dysfunction. Specifically, alterations in glutamate receptors, including N-methyl-D-aspartate (NMDA) and alpha-amino-3-hydroxy-5-methyl-4-isoxazolepropionic acid (AMPA) receptors, disrupt excitatory neurotransmission and compromise synaptic plasticity, which is vital for learning and memory.

GABAergic signaling, particularly through extrasynaptic GABA receptors (GABAARs and GABABRs), is also significantly altered in aging and AD, affecting neuronal inhibition (Vinod et al. 2017; McQuail et al. 2015). In the hippocampus, a reduction in GABA synthesis—due to decreased expression of glutamic acid decarboxylase (GAD)—leads to increased excitability of neurons. This heightened excitability can contribute to cognitive deficits, including impairments in memory retrieval and encoding. Conversely, in the aged PFC, an increase in tonic inhibition occurs due to elevated GABA production and decreased clearance mechanisms, which negatively impact working memory (McQuail et al. 2015).

The altered balance between excitatory and inhibitory signaling in these brain regions emphasizes the importance of synaptic dynamics in maintaining cognitive health. Region-specific changes in GABA signaling suggest that therapeutic strategies must be tailored to normalize inhibitory dynamics differently in the hippocampus and PFC. For example, targeting specific GABA receptor subtypes may provide a promising approach to alleviate cognitive decline associated with aging and AD (McQuail et al. 2015). By enhancing inhibitory control where it is diminished and moderating excessive inhibition where it is heightened, such strategies could potentially restore synaptic balance and improve cognitive outcomes.

Astrocytes, which are cells that secrete various neurotransmitters and factors that regulate neuronal growth, play an important role in this process. Studies have shown that astrocytes from Alzheimer's disease patients have changes in genes related to glutamate receptors. Glutamate binds to the *N*-methyl-d-aspartate (NMDA) subtype of glutamate receptors. Almost all neurons in the CNS have NMDA receptors, and excessive activation of these receptors leads to excitotoxicity (Verkhratsky et al. 2023). AD also involves metabolic reprogramming of astrocytes, which may affect their ability to maintain balance in the nervous system. A mutation in the APOE4 gene, associated with the risk of AD, leads to disorders in energy transmission in the brain and negatively affects the functions of astrocytes and neurons (Qi et al. 2021). Metabolic abnormalities in astrocytes may lead to insufficient metabolic support for neurons, which ultimately leads to synaptic dysfunction and neurodegeneration.

#### Inflammation

While the exact pathophysiological mechanism of AD remains unclear, there seems to be a role for inflammation—a complex immune response that systematically works to eliminate invading pathogens, respond to traumatic events, or counteract injurious agents These agents can originate from within the organism itself, like a necrotic cell, or externally, such as viruses and bacteria. Inflammation can manifest as either acute or chronic (Zhang et al. 2015). Neuroinflammation is acknowledged as a significant factor in the onset of chronic neurodegenerative diseases, prominently exemplified by AD.

Several studies have demonstrated the presence of various inflammatory markers in the brains of individuals with Alzheimer's disease, including elevated levels of inflammatory cytokines such as interleukin-1 beta (IL-1β), interleukin-6 (IL-6), and tumor necrosis factor-alpha (TNF-α), as well as chemokines such as such as IL-8 (interleukin-8), along with the accumulation of activated microglia in the affected regions, particularly the hippocampus, cerebral cortex, amygdala, and basal forebrain, which are critical for memory and cognitive functions. (Walker et al. 2019a, b; Lee et al. 2010; Kinney et al. 2018; Moore et al. 2002). Epidemiological research indicates that prolonged use of non-steroidal anti-inflammatory drugs can mitigate AD progression and postpone its onset, underscoring the significant association between neuroinflammation and AD pathogenesis (Lee et al. 2010).
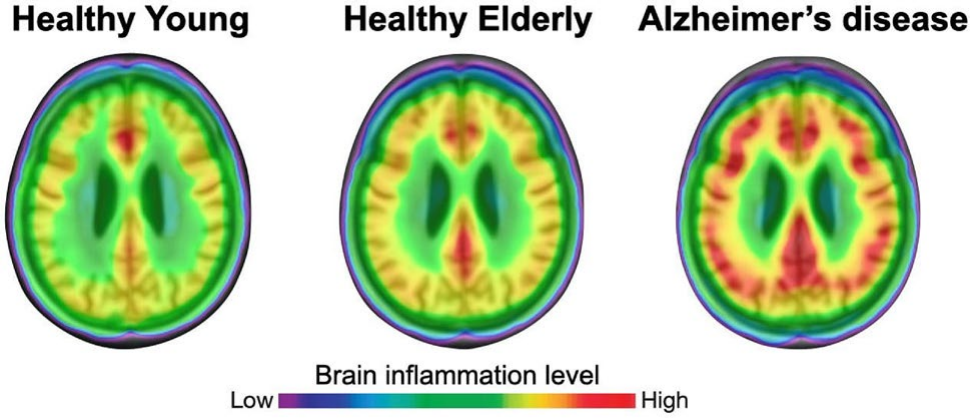


Systemic inflammation, characterized by elevated levels of pro-inflammatory cytokines and chemokines in the bloodstream, can arise from various triggers including infection, chronic illnesses, and both physical and psychological stress. Additionally, it may manifest independently of these conditions, stemming from subtle processes like cellular senescence (Walker et al. 2019a, b). Walker, Hoogeveen, and Gottesman’s discoveries offer a valuable understanding of the chronological connection between inflammation and negative neurocognitive consequences. They propose that systemic inflammation occurring many years before the usual onset age of dementia could accelerate the advancement of cognitive deterioration and neurodegenerative processes (Walker et al. 2019a, b). A significant mechanism by which inflammation is thought to contribute to neurodegenerative diseases involves the alteration of metabolites resulting from the breakdown of the essential amino acid tryptophan, known collectively as "kynurenines." This breakdown generates various bioactive intermediate metabolites, such as kynurenic acid (KA), 3-hydroxykynurenine (3HK), 3-hydroxyanthranilic acid (3HA), and quinolinic acid (QA), which can directly influence neurotransmitter receptors, impact redox processes, and regulate the activity of immune cells (Schwarcz et al. 2012).

#### Mitochondrial Dysfunction

The mitochondria stand out as the powerhouse of the cell, playing a pivotal role in meeting its energy demands. They oversee cellular energy metabolism and govern pathways related to cell apoptosis (McBride et al. 2006). Under normal circumstances, the equilibrium between fission and fusion reactions ensures the proper shape and size of mitochondria. However, in AD, this equilibrium is disrupted by the abnormal expression of genes like Dynamic-related protein-1 (Drp-1), mitochondrial fission-1 (Fis-1), and fusion proteins (Mfn1, Mfn2, and Opa1). These genes are essential for maintaining the regular functionality of mitochondria (Reddy et al. 2011). Additionally, the reduced levels of Drp-1 observed in AD are implicated in irregular distribution, altered morphology, and dysregulation of mitochondrial division (Wang et al. 2008). In AD patients, β-amyloid plaques often accumulate within mitochondria and neuronal synapses. Research indicates that the deposition of these plaques disrupts the usual forward and backward transport of mitochondria in hippocampal neurons, ultimately resulting in synaptic degeneration in the brains of individuals with AD (Bhatia et al. 2022). Additionally, hyperphosphorylated tau can impede mitochondrial transport along axons, disrupting their distribution and exacerbating energy deficits in neurons. This interference has been observed in various in vitro and in vivo models of Alzheimer's disease, including transgenic mouse models that express human tau and amyloid-beta (Aβ). As mitochondria are the primary source of reactive oxygen species (ROS), the increased oxidative imbalance and elevated ROS levels in AD patients serve as additional evidence of mitochondrial dysfunction in AD pathology. Studies have shown that plasma levels of endogenous antioxidants such as bilirubin, uric acid, and albumin are lower in AD patients, indicating an oxidative imbalance in their brains. This imbalance results in a significant rise in cerebral lipid peroxidation, as well as oxidation of proteins, DNA, and RNA, ultimately leading to neuronal death (Wang et al. 2014). However, Aβ and tau proteins contribute to mitochondrial dysfunction by directly interacting with mitochondrial components, disrupting bioenergetics, and impairing calcium homeostasis (Fišar 2022). Their presence leads to decreased ATP production and increased reactive oxygen species (ROS), which together enhance oxidative stress and promote neuronal damage. Furthermore, excessive calcium influx into mitochondria activates apoptotic pathways, exacerbating neurotoxicity and cell death homeostasis (Fišar 2022). This synergistic toxicity from Aβ and tau not only accelerates neurodegeneration but also influences pathways related to neuroinflammation and impaired cellular transport homeostasis (Fišar 2022).

What' s more, mitochondrial dysfunction significantly characterizes the aging process, as aging mitochondria become less efficient in energy production, accumulate oxidative damage, and exhibit impaired mitochondrial quality control (MQC), leading to increased cellular dysfunction (Guo et al. 2023). The decline in MQC involves an imbalance in mechanisms like proteostasis, dynamics, and mitophagy, where proteins such as PINK1 and Parkin lose effectiveness, resulting in the accumulation of damaged mitochondria that further exacerbates oxidative stress and cellular damage (Huang et al. 2020; Guo et al. 2023). This imbalance in MQC processes can accelerate cellular aging, contributing to metabolic dysfunction and age-related diseases.

Although it is uncertain whether mitochondrial dysfunction occurs before the onset of pathological protein changes, all these studies highlight the key role of mitochondria in the development of AD. Enhancing mitochondrial function presents promising opportunities for AD treatment, with antioxidants like resveratrol and curcumin showing benefits for memory deficits. Resveratrol activates SIRT1, promoting mitochondrial biogenesis and neuronal survival, while reducing amyloid-beta accumulation and inflammation in transgenic mouse models, which improves cognitive function (Gomes et al. 2018). Similarly, curcumin's potent antioxidant properties protect brain mitochondria from oxidative stress, mitigating damage linked to neurodegenerative disorders (Mishra and Palanivelu 2008).

#### Autophagy and Protein Removal

Autophagy, a process reliant on lysosomes, is crucial for maintaining cellular balance. It involves the degradation and recycling of organelles and proteins to generate energy. Consequently, impaired autophagy is implicated in the buildup of harmful proteins in the brain, potentially contributing to AD (Uddin et al. 2018). Autophagy is categorized into three types according to how intracellular components are delivered to lysosomes for degradation: microautophagy, chaperone-mediated autophagy, and the most prevalent form, macroautophagy. In healthy mammalian cells, there is a naturally low level of autophagy occurring at baseline. This basal autophagic activity is primarily responsible for maintaining intracellular balance by recycling proteins and organelles. In post-mitotic neuronal cells, this basal autophagy is particularly crucial, likely because these cells cannot dilute harmful components through cell division (Funderburk et al. 2010). Various stressors like nutrient deprivation, hypoxia, or inflammation can boost autophagic activity. This heightened autophagy plays roles in both physiological processes and pathological conditions, such as cell death, elimination of intracellularly invading microorganisms, and suppression of tumors. Conversely, decreased autophagic capability is linked to aging (Uddin et al. 2018). The decrease in autophagy contributes to the build-up of dysfunctional proteins and organelles, impairing cellular function and promoting the aging process (Levine and Klionsky 2004). In the context of Alzheimer's disease, autophagy exhibits a nuanced behavior that can differ at various stages of the disease. In the initial stages of AD, autophagy may be activated as a compensatory response to the accumulation of amyloid-beta (Aβ) plaques and tau tangles. This increased autophagic activity is an attempt to clear toxic proteins, potentially mitigating some early pathological effects (Nixon 2013). As AD progresses, the efficiency of autophagy may decline. The presence of aggregated proteins can hinder the autophagic process, leading to a vicious cycle where impaired autophagy results in further protein accumulation, thereby exacerbating neurodegeneration. In later stages, this decline is associated with increased neuronal death and cognitive decline (Nixon 2013; Yin et al. 2016). It’s essential to note that autophagy's role in AD can vary significantly among individuals and may depend on genetic factors, environmental influences, and the presence of other comorbidities.

According to a study by Nilsson et al. (2013), Aβ peptides, implicated in AD pathology, are released from neurons through an autophagy-dependent process. The study suggests that the buildup of intracellular Aβ plaques is harmful to brain cells, contributing to the development of Alzheimer's pathology. Removing these aggregates or preventing their formation are proposed therapeutic strategies for treating such disorders. Autophagy serves as a primary mechanism for degrading abnormal proteins, suggesting that leveraging autophagy mechanisms could aid in eliminating these aggregates. Several drugs that stimulate autophagy, such as carbamazepine and latrepirdine, have shown promising therapeutic potential for treating AD in clinical trials (Uddin et al. 2018).

## Anti-Aging Interventions in Alzheimer’s Disease

### Pharmacological interventions

#### Inhibitor of Amyloid-β Protease

The hypothesis that the accumulation of Aβ is at least partially due to age-related decline in Aβ degradation provides a credible mechanism that may explain a significant portion of AD cases. Nearly all individuals accumulate Aβ in the brain with age, suggesting that Aβ deposition may be an inevitable consequence of aging, which in turn may impose fundamental constraints on human brain health (Funato et al. 1998). Therefore, there is growing interest in the evolution of pharmacological inhibitors of proteases, especially β- and γ-secretases, responsible for the production of Aβ from βAPP (Zhang et al. 2023; Rogers et al. 2012).

BACE1 (beta-site amyloid precursor protein cleaving enzyme 1) is the protease responsible for the initial step in the production of amyloid-beta (Aβ) from amyloid precursor protein (APP) (Vassar et al. 1999). Inhibiting BACE1 as a prospective research direction in anti-aging therapy may be significantly important in preventing neuronal damage and cognitive dysfunction characteristic of age-related diseases (Zhang et al. 2023). LY2886721 was the first BACE inhibitor with improved brain penetration, and promising Phase 1 results from 2012, announced by Eli Lilly, included reduced levels of Aβ-40 and Aβ-42 in cerebrospinal fluid of mice and dogs. However, Phase 2 trials were halted due to adverse events related to elevated liver parameters (May et al. 2015). Elenbecestat (E2609), a BACE1 inhibitor, demonstrated reduced levels of Aβ protein in the brains, cerebrospinal fluid, and serum in preclinical studies with rats and guinea pigs. Results from numerous Phase 1 trials confirmed that elenbecestat was well-tolerated at all tested single doses. However, due to an unfavorable risk–benefit ratio, the Phase 3 study was discontinued (Madrasi et al. 2021). Several other BACE inhibitors, including atabecestat (Sperling et al. 2021), lanabecestat (Wessels et al. 2020), and umibecestat (Neumann et al. 2018), progressed to late-stage clinical trials, but none received final approval. Developing effective BACE1 inhibitors faces challenges due to structural similarities with other aspartyl proteases like BACE2, pepsin, renin, cathepsin D, and E, posing obstacles to achieving selective inhibition without side effects (Zhang et al. 2023).

Gamma-secretase inhibitors (GSI) also represent a promising therapy, reducing levels of Aβ. However, the general mechanism of action of GSIs led to serious side effects by blocking protein processing, including Notch, hence the increasing need to discover agents with selective action (Zhang et al. 2023). Avagacestat, a specific gamma-secretase inhibitor, lowered Aβ levels without affecting Notch, but Phase 2 studies were halted due to undesirable gastrointestinal and dermatological effects (Coric et al. 2015). In a study conducted by Rogers et al. using an AD in vivo model, the gamma-secretase modulator EVP-0015962 reduced levels of toxic Aβ-42 peptides without impacting Notch signaling and improved memory deficits in mice, making it a promising approach in AD treatment (Rogers et al. 2012).

In spite of encouraging outcomes in early clinical trials, the development of effective BACE1 and GSI inhibitors faces several challenges (Zhang et al. 2023; May et al. 2015; Madrasi et al. 2021; Sperling et al. 2021; Wessels et al. 2020; Neumann et al. 2018). These challenges encompass the need for very specific inhibition to target the intended pathways without affecting others, as well as the crucial need to minimize and manage potential side effects. Research into innovative approaches, such as γ-secretase modulators, demonstrates significant potential in the fight against Alzheimer's disease (Zhang et al. 2023). These modulators are designed to selectively reduce levels of toxic Aβ-42 peptides, which are implicated in the neurodegenerative processes characteristic of the disease. By lowering the concentration of these harmful peptides, γ-secretase modulators not only aim to halt or slow the progression of Alzheimer's but also have shown promise in ameliorating memory deficits (Zhang et al. 2023).

#### Autophagy Enhancement

Autophagy is a lysosomal degradation process that protects cells from stress, allowing for the removal of damaged cellular organelles, pathogens, and misfolded proteins (Redmann et al. 2016). Autophagy plays a key role in the pathogenesis of many diseases, and its enhancement is suggested to be beneficial in treating infections, metabolic disorders, neurodegenerative diseases, and even cancer. As previously mentioned, age-related dysfunction of autophagy may contribute to the etiology of AD by promoting the accumulation of toxic proteins such as Aβ and tau (Uddin et al. 2018). Furthermore, autophagy significantly influences the aging process, with i*n vitro and *in vivo studies showing that increased expression of autophagy-related genes promotes longevity (Hansen et al. 2018).

Currently, many studies are focused on exploring therapies that could enhance autophagy processes. Some of these studies are investigating existing substances used in the treatment of other diseases, which have the potential to increase autophagy. One such drug is metformin, used in type 2 diabetes treatment, which has been found to inhibit mTOR activity and protein synthesis, leading to decreased metabolic pathway activity and induction of autophagy (Shi et al. 2012). In a study by Spilman et al. (2010) using a mouse model of AD, blocking mTOR signaling with rapamycin alleviated cognitive deficits and reduced amyloid pathology accumulation. These findings suggest that the action of rapamycin may be associated with autophagy stimulation in brain cells (Spilman et al. 2010). Additionally, Li et al. (2013) reported that autophagy induced by the antiepileptic drug carbamazepine protects against memory impairments and reduces Aβ levels in a mouse model of AD (Li et al. 2013). A meta-analysis has shown that lithium significantly reduces cognitive decline compared to placebo, which may be related to its autophagy-inducing activity independent of mTOR (Sakar et al. 2005; Matsunaga et al. 2015). Furthermore, resveratrol, curcumin, and spermidine used in dietary supplements can also stimulate autophagy, with beneficial effects on the cardiovascular and nervous systems (Shakeri et al. 2019; Eisenberg et al. 2016; Kodali et al. 2015).

The accumulation of amyloid-beta (Aβ) peptides is a key factor in the development of AD, driving the search for therapeutic strategies that either reduce Aβ production or enhance the breakdown of neurotoxic compounds. Traditional approaches using protease inhibitors to prevent amyloid plaque formation have encountered significant challenges due to their lack of selectivity, leading to numerous side effects such as gastrointestinal issues, immunological reactions, metabolic changes, organ toxicity and allergic reactions (Beher et al. 2005).

Recent discoveries by Tian et al. (2011) have opened new avenues for AD treatment through the use of SMER28, an autophagy activator. This compound enhances the breakdown of Aβ and the amyloid precursor protein C-terminal fragment (APP-CTF) in vitro without causing negative effects on other proteins like Notch and APLP1 (amyloid precursor-like protein 1) (Tian et al. 2011). This selective activation of autophagy offers a promising therapeutic strategy for age-related diseases like AD, aiming at the fundamental mechanisms of protein buildup.

In conclusion, studies on autophagy in the context of AD are yielding promising results. Stimulating autophagy could serve as a potential therapeutic approach, not only by preventing the accumulation of toxic proteins such as Aβ and tau but also by mitigating cognitive deficits associated with AD. However, further research is essential to ascertain whether autophagy activation alone is sufficient to counteract the aging processes and effectively treat AD. Continued exploration in this field may pave the way for new, more effective treatments, offering hope for patients suffering from this debilitating condition.

#### Plasma Transfusion

Research into plasma transfusion from young individuals as a strategy to combat aging is garnering significant attention within the scientific community. This concept hinges on the potential benefits of infusing young, healthy plasma components into the bodies of older individuals, aiming to rejuvenate and improve their physiological functions (Scudellari et al. 2015). By introducing these youthful factors, researchers hope to mitigate the effects of aging and promote overall health and longevity. Parabiosis is an experimental research technique, primarily used in animals, involving the connection of blood circulation between two organisms, often one young and one older. This type of connection enables the study of the impact of interactions between organisms on biological processes such as tissue regeneration, aging, or disease development (Castellano et al. 2019). In a preclinical study conducted by Middeldorp and colleagues, a potentially beneficial impact of heterochronic parabiosis in the context of Alzheimer's disease was observed. The study found that blood circulation from young mice to mice with AD did not affect beta-amyloid deposition or microglial activation; however, it reversed synapse loss and normalized gene expression related to neuronal signaling pathways in the hippocampus. Additionally, repeated administration of plasma from young healthy mice to AD mice led to improvement in spatial working and associative memory (Middeldorp et al. 2016).

In a phase 2 clinical trial, it was found that systemic administration of growth hormone-releasing hormone (GHRH), present in the blood of young individuals, appears to beneficially affect cognitive function in healthy older humans and adults with mild cognitive impairment (MCI) (Baker et al. 2012). Additionally, TIMP2 (Tissue Inhibitor of Metalloproteinases 2) is elevated in young mice and humans. It was discovered that systemic administration of TIMP2 enhances synaptic plasticity and improves learning and memory functions in older mice (Castellano et al. 2017). Furthermore, osteocalcin (OCN) is identified as another critical component of young plasma activity, and systemic treatment with OCN improves cognitive functions in older mice, acting in part through the GPR158 receptor (Khrimian et al. 2017).

However, the anti-aging method of plasma transfusion does have certain limitations. First, it is crucial to determine the optimal age range and health status of donors whose plasma can provide the most beneficial effects to older recipients, and vice versa. Additionally, identifying the precise volume and frequency of transfusions necessary to achieve therapeutic benefits without causing adverse effects is essential. Potential risks include lung damage, circulatory overload, allergic reactions, and infections (Pandey et al. 2012).

Moreover, the application of plasma transfusion in humans remains controversial. The ethical implications, long-term safety, and overall efficacy of this approach are subjects of ongoing debate and require extensive research before plasma transfusion can be considered a viable medical therapy (Hofmann 2018). Comprehensive clinical trials and rigorous scientific studies are needed to address these concerns and to establish standardized protocols that maximize benefits while minimizing risks.

#### Senolitics

Aging cells are characterized by irreversible cell cycle arrest and resistance to apoptosis—the process of programmed cell death. Furthermore, senescent cells (SCs) adopt a senescence-associated pro-inflammatory secretory phenotype (SASP), which includes cytokines, chemokines, proteases, and other factors that promote inflammation and tissue damage. SASP can be induced by various stressors, both intracellular (e.g., DNA damage) and extracellular (e.g., UV radiation) (Bussian et al. 2018). Therefore, drugs called senolytics are becoming more and more popular.

Senolytics are pharmacological compounds that selectively induce the death of senescent cells. They work by targeting specific signaling pathways and survival mechanisms of SCs that differ from those in healthy cells (Zhu et al. 2015). Research indicates that eliminating SC with senolytics such as dasatinib and quercetin may offer a promising therapeutic approach. Dasatinib, as a tyrosine kinase inhibitor, interacts with p53 and inhibits plasminogen activator inhibitor type 2 (PAI-2), while quercetin, a natural flavonoid, acts on the PI3K (phosphatidylinositol 3-kinase) and HIF-1α (hypoxia-inducible factor 1-alpha) pathways. The joint action of these compounds may lead to the protection of neurons against damage, improvement of microcirculation, and reduction of inflammation in the brain, which potentially translates into slowing down the aging process and improving cognitive functions in old age (Zhu et al. 2015). Additionally, Navitoclax is a drug that targets protein families associated with apoptosis (programmed cell death), specifically proteins in the Bcl-2 family (Zhu et al. 2016). A study by Zhu (2016) showed that navitoclax acted as a senolytic agent in human umbilical vein endothelial cells that were cultured in laboratory conditions for an extended time, while it was not effective against human fat cell progenitors. This study suggests that the effectiveness of navitoclax as a senolytic agent may depend on the cell type and its specific characteristics, such as the origin or degree of aging (Zhu et al. 2016). Another promising compound is piperlongumine, derived from the *Piper Longum* plant (Makhov et al. 2014). Studies have shown that piperlongumine effectively reduces the activity of the Akt/mTOR pathway by generating ROS, which promotes cellular autophagy (Makhov et al. 2014). These findings suggest that piperlongumine may be useful in the treatment of aging and AD by modulating autophagy and signaling pathways. Potential therapies based on autophagy inhibitors may increase the efficiency of eliminating senescent cells and improve cognitive functions in AD (Makhov et al. 2014). Moreover, research by Pal et al. suggests that treatment of A431 cells with fisetin inhibited growth and induced apoptosis (Pal et al. 2013). These effects of fisetin result from G2/M arrest, modulation of Bcl2 family protein expression, loss of mitochondrial membrane potential, caspase activation, and PARP protein cleavage (Pal et al. 2013). In the context of aging and AD, these mechanisms may be important because these processes are associated with the mitochondrial dysfunction and apoptosis disorders observed in these conditions (Pal et al. 2013).

Senolytics, such as dasatinib (Zhu et al. 2015), quercetin (Zhu et al. 2015), navitoclax (Zhu et al. 2016), pipelongumine (Makhov et al. 2014), and fisetin (Pal et al. 2013), hold promising therapeutic potential in targeting and eliminating senescent cells. These drugs work by modulating specific signaling pathways that regulate cell survival and apoptosis, thereby improving cognitive functions and potentially slowing down the aging process and neurodegenerative diseases. However, it is essential to consider the diverse cellular environments and biological contexts when evaluating the effectiveness of senolytic drugs. Different cell types may respond differently to these treatments, and the overall impact can vary based on the specific disease conditions or aging-related processes. Therefore, careful consideration of these factors is crucial to optimize the therapeutic benefits of senolytics and minimize potential risks.

### Lifestyle Interventions

#### Sleep

Sleep plays a key role in the aging process of cells. During sleep, the body goes through various phases that are necessary for DNA repair, elimination of damaged cells, and tissue regeneration (Bellesi et al. 2016). Insufficient sleep leads to increased oxidative stress and inflammation, which accelerates the aging process of cells and impairs their functioning (Coulson et al. 2022). A notable feature of aging is the decline in sleep quality, particularly a decrease in slow wave sleep (SWS) during non-rapid eye movement (NREM) stages (Liguori et al. 2014). This decline in deep, restorative sleep is a common feature observed in older adults and is associated with various health issues. Additionally, there is often a reduction in rapid eye movement (REM) sleep, the phase associated with dreaming, which is critical for memory consolidation and emotional regulation. The reduction of REM sleep has been linked to increased vulnerability to cognitive decline and emotional dysregulation in aging populations (Bliwise 2004; Pace-Schott and Spencer 2011).

Furthermore, individuals with AD often experience disrupted sleep patterns, which can exacerbate cognitive decline and other symptoms of the disease (Lloret et al. 2020). Sleep disturbances in AD patients are multifaceted, involving difficulties in falling asleep, maintaining sleep, and altered sleep architecture, including reduced REM sleep. The loss of REM sleep in AD patients is particularly concerning as it may contribute to the rapid progression of the disease by impairing memory consolidation and increasing neuroinflammation (Lim et al. 2013). These disruptions not only affect the patients' quality of life but also contribute to the progression of cognitive impairment (Lloret et al. 2020). The study by Liguori et al. (2014) showed a significant correlation between high levels of tau and Aβ proteins and sleep disorders (Liguori et al. 2014). In turn, a study conducted by Osorio et al. (2015) reports that individuals suffering from sleep apnea, a condition often associated with disrupted REM sleep, exhibited symptoms of AD and MCI more quickly. These findings suggest that proper sleep may reduce the severity of pathology associated with Alzheimer's disease (Osorio et al. 2015).

The mechanism explaining these discoveries could be the fact that during NREM, there is decreased oxygen consumption and active replenishment of ATP levels (Braun et al. 1997). In the waking state, the consumption of oxygen, ATP and glucose increases, leading to a higher level of metabolic load and consequently to oxidative homeostasis disruption and neurotoxicity (Braun et al. 1997; Villafuerte et al. 2015; Coulson et al. 2022). Therapeutic strategies that aim to improve sleep quality and quantity may potentially mitigate cognitive decline and improve overall health outcomes in aging populations and AD patients.

#### Exercise

**Fig. 4:**
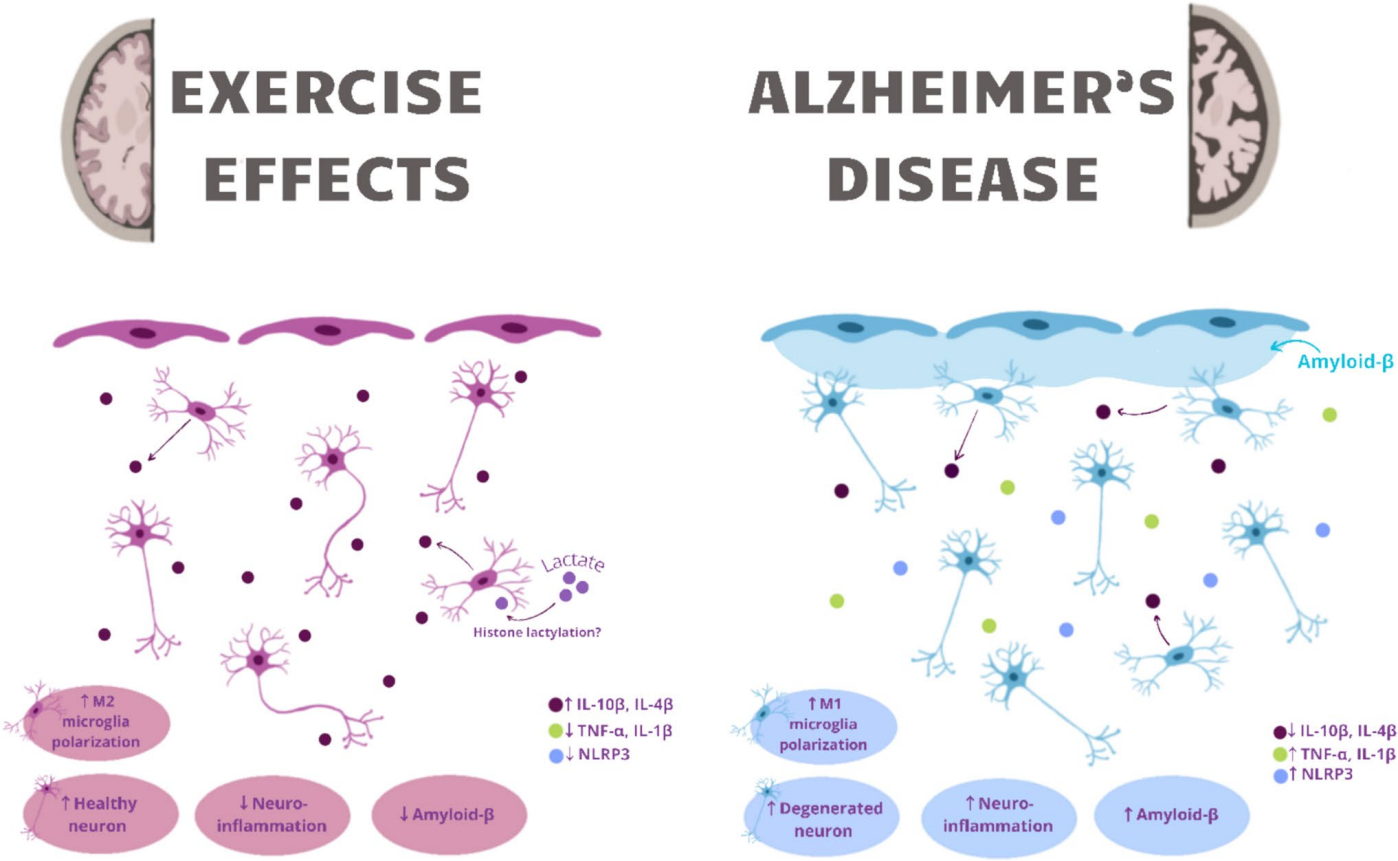
Exercise benefits on Alzheimer's disease

The physiological aging process largely determines the slowing down and gradual limitation of activity in older people. However, lack of physical activity is a factor determining the deterioration of cognitive functions both in the aging process and in neurodegenerative diseases (Norton et al. 2014). Currently, there is growing evidence suggesting that regular exercise may have a preventive effect and potentially slow down the development of AD (Norton et al. 2014; Tolppanen et al. 2015; Okonkwo et al. 2014) (Fig. 4). Research by Okonkwo et al. (2014) suggests that increased physical activity is linked to reduced levels of amyloid-beta in the bloodstream, a key protein associated with AD pathology.

Moreover, brain exercises are increasingly recognized as an effective anti-aging therapy. Regularly stimulating the mind through various intellectual activities, such as solving puzzles, reading, learning new skills, and participating in logic games, can significantly improve cognitive functions (Wilson et al. 2002). Research shows that engaging the brain in diverse tasks not only helps maintain mental acuity but can also delay the onset of neurodegenerative diseases such as AD (Wilson et al. 2002; Lin et al. 2018). Regular physical activity, particularly aerobic exercise, has been linked to improved cognitive function, a reduced risk of cognitive decline, and increased neural adaptability (Lin et al. 2018). Aerobic exercise fosters the growth of new blood vessels (angiogenesis) and stimulates the release of key neurotrophic factors such as IGF-1, BDNF, and VEGF. These factors not only improve cerebral blood flow but also play a crucial role in supporting neural function by promoting neurogenesis, enhancing synaptic plasticity, and protecting neurons from injury. Moreover, these factors play a key role in neuroprotection by facilitating the clearance of beta-amyloid and mitigating tau protein hyperphosphorylation, both of which are central to the pathology of Alzheimer's disease (Paillard et al. 2024). This dual impact on both systemic and brain-specific markers of AD pathology highlights the potential of physical activity as a non-pharmacological intervention for reducing the risk of AD and improving overall brain health.

Despite these promising findings, further research is required to establish cause-and-effect relationshipsand to identify the optimal types, intensities, and durations of exercise needed to achieve maximum cognitive benefits. Integrating regular physical activity into daily routines may offer a cost-effective and accessible strategy to promote brain health and mitigate the onset and progression of AD.

#### Diet

Various dietary elements and foods have been identified as either risk factors or protective factors for the development of dementia and Alzheimer’s disease. These elements include fats, fatty acids, antioxidants, homocysteine/methionine, vitamins, and alcohol (Petot and Friedland 2015). Petot and Friedland (2015) suggest that brain diseases associated with aging are not solely the result of pathogenic processes but also stem from the failure of protective mechanisms. Moreover, diet plays a crucial role in the effectiveness of these protective mechanisms.

Among healthy dietary patterns, the Mediterranean diet (high consumption of whole grains, fruits, vegetables, legumes, and olive oil, moderate intake of cheese and fish, and limited intake of meat, particularly red and processed meat) has been linked to beneficial effects on various health outcomes, including cognitive function. A meta-analysis by Nucci et al. (2024) demonstrated that simply improving one's diet by adhering to the Mediterranean diet can significantly reduce the risk of developing dementia.

Cardiovascular risk factors like hypertension, abdominal obesity, dyslipidemia, and type 2 diabetes play a significant role in increasing the risk of dementia (Kim et al. 2021). These factors contribute to chronic inflammation and metabolic problems such as insulin resistance and hyperinsulinemia, which can damage the brain. Research indicates that high adherence to the Mediterranean diet can reduce various inflammation biomarkers associated with the onset of AD, including pro-inflammatory cytokines like interleukin-1β (IL-1β), interleukin-6 (IL-6), and tumor necrosis factor-alpha (TNF-α) (Bonaccio et al. 2023).

The diet’s ability to lessen chronic inflammation is linked to its rich supply of bioactive compounds, such as vitamins, minerals, phytochemicals, and essential fatty acids, which have potent antioxidant and anti-inflammatory effects (Lourida et al. 2013). Diet and inflammation are closely linked, significantly influencing neurological diseases like Alzheimer's, Parkinson's, multiple sclerosis, schizophrenia, bipolar disorder, and depression. Key dietary factors regulate inflammation, with whole foods—fruits, vegetables, whole grains, and lean proteins—associated with lower inflammatory markers such as C-reactive protein (CRP) and interleukin-6 (IL-6) (Kurowska et al. 2023). According to Kurowska et al. (2023), numerous studies indicate that the ketogenic diet has a beneficial effect on memory in patients with early AD or mild cognitive impairment. This suggests that fats may significantly improve individual patient outcomes.

### Gene Therapy

Gene therapy holds immense promise for providing therapeutic benefits to millions suffering from neurodegenerative diseases through a variety of approaches. The success of gene therapy in treating neurodegenerative diseases hinges on a deep understanding of the underlying pathogenesis of these conditions and achieving accurate regulation of when and where genes are expressed. One significant challenge lies in ensuring comprehensive transduction of the targeted area while preventing unintended spread to adjacent regions or perivascular spaces (Sudhakar et al. 2019).

Neurodegenerative diseases are characterized by the gradual accumulation of malfunctioning proteins within cells, ultimately leading to cell death. Current treatments only offer symptomatic relief, as they do not address the root causes of these diseases. As the disease progresses, the efficacy of pharmacological treatments diminishes, even with higher doses of medication, resulting in an unfavorable increase in the side effect-to-benefit ratio. Intracerebral drug delivery, particularly through gene therapy, represents a promising approach to overcome these limitations in medical management (Sudhakar et al. 2019). By directly targeting the affected areas of the brain, gene therapy can potentially correct genetic mutations, enhance neuroprotection mechanisms, stimulate neuronal regeneration, and alleviate symptoms associated with neurodegenerative diseases.

The multifactorial nature of AD presents numerous potential targets for gene therapy. These include neurotrophic growth factors like nerve growth factor and brain-derived neurotrophic factor, enzymes that degrade Aβ such as neprilysin, endothelin-converting enzyme, and cathepsin B, as well as AD-associated apolipoprotein E (Nilsson et al. 2010).

However, the field of gene therapy for neurodegenerative diseases faces several challenges, including the need for safe and effective gene delivery systems, long-term gene expression, and the potential for immune responses. Continued research and clinical trials are essential to validate the safety and efficacy of gene therapy approaches and to pave the way for their widespread application in clinical practice.

## Conclusions

In conclusion, anti-aging interventions in AD represent a beacon of hope in the quest for effective treatments and preventive strategies against this devastating disease. Recent advancements in research have unveiled promising avenues for intervention, ranging from lifestyle modifications to pharmacological interventions and emerging therapies targeting specific molecular pathways implicated in aging and AD pathogenesis. Lifestyle interventions encompassing regular exercise, cognitive stimulation, dietary modifications, and social engagement have shown potential in promoting brain health and resilience against age-related cognitive decline. Pharmacological interventions targeting amyloid-beta and tau protein pathology, neuroinflammation, mitochondrial dysfunction, and synaptic loss hold promise as potential disease-modifying therapies. Emerging approaches leveraging gene therapy, stem cell transplantation, and novel drug delivery systems offer innovative strategies for combating AD and rejuvenating aging brains.

However, the translation of these discoveries from bench to bedside remains a formidable challenge, requiring rigorous clinical trials, robust biomarkers for early detection and monitoring, and personalized treatment approaches tailored to individual disease trajectories.

As we confront these challenges, we must remain unwavering in our dedication to expanding our knowledge of Alzheimer's disease and the aging process. This entails not only enhancing our understanding of the underlying mechanisms but also ensuring that emerging interventions are accessible to all individuals, regardless of socioeconomic status or geographical location. Moreover, it is vital to cultivate collaboration across diverse fields and disciplines to synergistically address the complexities of AD and aging. By collectively striving towards these goals, we can envision a future where the burden of Alzheimer's disease is significantly reduced, and aging populations worldwide can thrive with cognitive resilience and vitality.

## Data Availability

No datasets were generated or analysed during the current study.
